# Tuberculosis Disparity between US-born Blacks and Whites, Houston, Texas, USA^1^

**DOI:** 10.3201/eid1506.081617

**Published:** 2009-06

**Authors:** Jose A. Serpa, Larry D. Teeter, James M. Musser, Edward A. Graviss

**Affiliations:** Baylor College of Medicine, Houston, Texas, USA (J.A. Serpa, E.A. Graviss); The Methodist Hospital Research Institute, Houston (L.D. Teeter, J.M. Musser, E.A. Graviss)

**Keywords:** Mycobacterium tuberculosis, tuberculosis and other mycobacteria, epidemiology, bacteria, minorities, Texas, research

## Abstract

Tuberculosis (TB) rates in the United States are disproportionately high for certain ethnic minorities. Using univariate and multivariate analyses, we compared data for 1,318 US-born blacks with 565 US-born non-Hispanic whites who participated in the Houston TB Initiative (1995–2004). All available *Mycobacterium tuberculosis* isolates underwent susceptibility and genotype testing (insertion sequence *6110* restriction fragment length polymorphism, spoligotyping, and genetic grouping). TB in blacks was associated with younger age, inner city residence, HIV seropositivity, and drug resistance. TB cases clustered in 82% and 77% of blacks and whites, respectively (p = 0.46). Three clusters had >100 patients each, including 1 cluster with a predominance of blacks. Size of TB clusters was unexpectedly large, underscoring the ongoing transmission of TB in Houston, particularly among blacks.

## Abstract 2

An unusually high proportion of cases in Houston are caused by active transmission of endemic strains among US-born non-Hispanic blacks.

After the unprecedented resurgence in tuberculosis (TB) in the United States during 1985–1992, the annual incidence rate of TB steadily decreased from 1993 to 2007. However, this decline recently decelerated, raising concerns that progress toward eliminating TB is slowing. In 2007, a total of 13,293 TB cases (4.4 cases per 100,000 population) were reported in the United States, representing a 4.2% decline in incidence from 2006 (*1*). Despite this decline, TB incidence remains higher among certain racial/ethnic minorities, i.e., blacks, Hispanics, and Asians, than for non-Hispanic whites. For instance, US-born young blacks probably account for the largest number of secondary TB cases in the country (*2*). In addition, TB rates for non-Hispanic blacks continue to be 8 times greater than for non-Hispanic whites (*3*). Hispanics and non-Hispanic blacks also accounted for nearly three quarters (73.9%) of 11,480 TB cases in children reported during 1993–2001, of which more than half occurred in children <5 years of age (*4*).

These findings clearly suggest recent ongoing transmission of *Mycobacterium tuberculosis* in these ethnic groups rather than reactivation of latent infection. Recently, molecular epidemiologic studies, which estimate the proportion of clustered TB cases, have been used to support this hypothesis (*5*). For instance, a molecular epidemiologic study of TB in San Francisco during 1991–1992 found *M. tuberculosis* clustering in 191 (41%) of 471 patients studied that were distributed in 44 clusters. Hispanic ethnicity or black race, birth in the United States, and an AIDS diagnosis independently predicted clustering (*6*). Similarly, other population-based studies in New York City and in Tarrant and Harris counties in Texas found clustering of 36%–52% (*7*–*9*).

Molecular studies of clustering and identification of risk factors that contribute to these racial/ethnic disparities are crucial for efforts to eliminate TB in the United States. Although differences in socioeconomic status and access to the healthcare system among non-Hispanic blacks have been postulated as the main barriers to TB diagnosis and treatment in this group, further information is needed (*3*,*10*). We studied the traditional and molecular epidemiology of TB in US-born blacks enrolled in the Houston Tuberculosis Initiative (HTI) during 1995–2004.

## Materials and Methods

### Study Population

The study population comprised patients recruited through HTI, a population-based active surveillance and molecular epidemiologic study that enrolls persons with TB reported to the City of Houston Department of Health and Human Services and Harris County Public Health and Environmental Services. In this study, we examined data from TB patients enrolled by HTI who had been reported during October 1995–September 2004.

Study participants were interviewed by trained interviewers using a standardized questionnaire designed to gather details of participant demographics, living situation, transportation, travel and social contacts, tobacco and alcohol use, illicit drug use, sexual behavior, history of incarceration, and personal medical history. Clinical information was supplemented through record review of all available inpatient, outpatient, and public health medical records.

### Molecular Characterization of *M. tuberculosis*

We analyzed *M. tuberculosis* isolates with 3 molecular typing methods. First, isolates were characterized by an internationally standardized protocol of insertion sequence [IS] *6110* restriction fragment length polymorphism (RFLP) profiling (*11*) and analyzed with BioImage Whole Band Analysis version 3.2 software (BioImage, Ann Arbor, MI, USA). Second, for isolates with <5 IS*6110* copies, the spacer oligonucleotide type (spoligotype) was determined by using a commercially available kit (Isogen Bioscience BV, Maarsen, the Netherlands) according to the manufacturer’s instructions. A member of the Beijing family was defined as having an octal spoligotype pattern of 000000000003771 (previously designated as S1) (*12*). Third, isolates were assigned to a principal genetic group on the basis of polymorphisms at codon 463 of the *katG* gene, which encodes catalase peroxidase, and at codon 95 of the *gyrA* gene, which encodes the A subunit of DNA gyrase (*13*). Susceptibility testing was performed by using BACTEC 460 radiometric culture system (Becton Dickinson, Sparks, MD, USA), at the hospital or reference laboratories supplying isolates to the HTI (*14*).

### Definitions

We assigned race/ethnicity on the basis of self-report. *M. tuberculosis* isolates were defined as clustering if they satisfied 1 of the following criteria: 1) >2 isolates from the same principal genetic group with identical IS*6110* banding profiles containing >5 copies of IS*6110* or 2) isolates with identical IS*6110* banding profiles containing <4 copies of IS*6110* and sharing the same spoligotype and principal genetic group. We evaluated clusters H03 and H33 as a single cluster because they differ in only 1 band by IS*6110* RFLP.

We defined drug-resistant TB as an isolate resistant to at least 1 drug, including isoniazid, rifampin, ethambutol, pyrazinamide, or streptomycin. Multidrug-resistant TB was defined by resistance to isoniazid and rifampin, with or without resistance to other agents.

### Data Analysis

Questionnaire data were entered into a longitudinal database (initially Epi Info version 6.02b, Centers for Disease Control and Prevention, Atlanta, GA, USA; and subsequently Microsoft Access, Microsoft, Redmond, WA, USA) for storage and analysis. Statistical analyses were conducted with STATA version 8.0 SE (StataCorp., College Station, TX, USA). Descriptive analysis was initially conducted, and selected demographic, social, and clinical factors were studied by univariate logistic regression to test for differences between US-born blacks and US-born whites. We used US-born whites, as opposed to all others, as the comparison group because of the heterogeneity in the latter group, particularly in non–US-born Hispanics and Asians. Variables with p<0.2 in the univariate analysis were considered in the elaboration of a multivariate logistic regression model to identify with factors independently associated with black race in TB patients. Covariates were not included in the model if they substantially decreased the sample size because of missing data. In our final model, we used the variable injection drug use instead of drug use because of colinearity and a strong association with the former variable. Covariates that were no longer significant after adjustment for other significant variables in the full model were dropped from the final parsimonious model.

### Ethical Approval

Individual participants gave written informed consent before enrollment in the study. We obtained parental consent and patient assent for participants 13 to <18 years of age, and a parent served as a proxy for the interview of participants <13 years of age. Proxy permission and interview also were used for participants who had died or were not mentally capable of giving informed consent. The study was approved by the Institutional Review Board of Baylor College of Medicine, Houston, Texas, and affiliated hospitals, and the University of Texas Health Science Center–Houston, Committee for the Protection of Human Subjects.

## Results

### Study Population

A total of 4,312 persons with TB were reported in Harris County during October 1995–September 2004. Of those, 3,662 (85%) agreed to participate in the study and were interviewed by trained HTI personnel. The study population comprised 1,318 (36.0%) US-born blacks, 1,220 (33.3%) Hispanics, 545 (14.9%) US-born non-Hispanic whites, 463 (12.6%) Asians, 85 (2.3%) foreign-born blacks, 20 (0.5%) foreign-born non-Hispanic whites and 11 (0.3%) others. Of participants interviewed, 3,064 (84%) had a positive culture for *M. tuberculosis*; 2,806 (92%) of those isolates underwent molecular characterization. Interviewed participants were more likely to be younger, black, HIV-seropositive, and *M. tuberculosis* culture–positive than were persons who refused to participate or could not be located (p<0.01 for first 3 comparisons and p = 0.03 for the last comparison).

### Rates of TB during the Study Period

**Figure 1:**
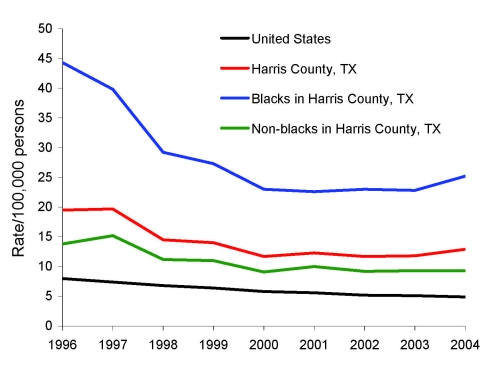
Tuberculosis rates, Houston Tuberculosis Initiative, Texas, 1995–2004.

Although the overall US incidence of TB decreased from 8.0 to 4.9 cases per 100,000 persons during 1996–2004, TB incidence in Harris County has remained fairly stable since 2000 after an initial decrease in 1996–1999 (Figure 1). Blacks consistently had the highest TB incidence in Harris County. In 2004, blacks accounted for approximately 56% of all TB cases among US-born participants despite representing only 18% of the Harris County population (Texas State Data Center and Office of the State Demographer, http://txsdc.utsa.edu/tpepp/txpopest.php).

### US-born Blacks versus US-born Whites

US-born blacks were more likely than US-born whites to be younger, unmarried, and unemployed; have less education; reside in the inner city, earn less income; use public transportation; have renal and extrapulmonary disease; be HIV seropositive, *M. tuberculosis* culture–positive, and part of a cluster; and have drug-resistant *M. tuberculosis* (Table 1).

**Table 1:** Univariate analysis of selected epidemiologic and clinical characteristics of participants in the Houston Tuberculosis Initiative, Texas, 1995–2004*

Variable	US-born black	US-born white	Hispanic	Asian	US-born black vs. white, OR (p value)
Demographics						
Age, y, mean (median)	42.5 (43.0)	48.2 (47.0)	36.9 (36.0)	45.1 (43.0)	0.98 (<0.001)	
Male sex	938 (71.2)	429 (78.7)	783 (64.2)	257 (55.5)	0.67 (0.001)	
Not married	1,091 (82.8)	412 (75.6)	742 (60.8)	202 (43.6)	1.56 (<0.001)	
Years of education, mean (median)	10.6 12.0	11.7 12.0	254 (20.8)	260 (56.3)	0.90 (<0.001)	
Employed	453 (34.4)	214 (39.3)	501 (41.1)	209 (45.1)	0.81 (0.045)	
Inner city residence†	538 (40.9)	198 (36.7)	384 (31.5)	40 (8.6)	1.19 (0.094)	
Household size, mean (median)	5.2 (3.0)	4.5 (2.0)	4.7 (4.0)	5.1 (4.0)	1.00 (0.454)	
Social history						
Income <$10K/year	554 (51.7)	161 (36.0)	466 (46.0)	78 (18.1)	1.90 (<0.001)	
Use of public transportation	664 (50.4)	177 (32.5)	363 (29.8)	40 (8.6)	2.11 (<0.001)	
Current homelessness	129 (9.8)	77 (14.2)	32 (2.6)	4 (0.9)	0.66 (0.007)	
Stay in a shelter‡	132 (10.0)	65 (11.9)	17 (1.4)	5 (1.1)	0.82 (0.222)	
Current smoking	745 (56.7)	388 (71.5)	279 (22.9)	80 (17.3)	0.52 (<0.001)	
Alcohol abuse§	601 (45.8)	311 (57.4)	251 (20.6)	50 (10.8)	0.63 (<0.001)	
Drug use‡	351 (26.7)	129 (23.7)	110 (9.0)	4 (0.9)	1.17 0.179)	
Injection drug use‡	25 (1.91)	25 (4.6)	9 (0.74)	1 (0.22)	0.40 (0.002)	
Same-sex sexual behavior	129 (9.9)	91 (17.0)	69 (5.7)	2 (0.4)	0.54 (<0.001)	
Commercial sex	168 (13.1)	67 (12.6)	52 (4.3)	2 (0.4)	1.05 (0.758)	
Prison history	281 (21.4)	92 (16.9)	49 (4.0)	5 (1.1)	1.34 (0.026)	
Medical history						
Known TB exposure	502 (38.1)	222 (40.7)	375 (30.7)	86 (18.6)	0.88 (0.222)	
Previous TB	112 (8.5)	55 (10.1)	56 (4.6)	41 (8.9)	0.83 (0.274)	
Asthma	85 (6.4)	28 (5.1)	40 (3.3)	12 (2.6)	1.27 (0.282)	
Diabetes	168 (12.7)	61 (11.2)	224 (18.4)	62 (13.4)	1.16 (0.353)	
Renal disease	76 (5.8)	15 (2.8)	34 (2.79)	21 (4.54)	2.16 (0.007)	
HIV seropositivity	364 (27.6)	109 (20.0)	111 (9.1)	8 (1.7)	1.52 (0.001)	
Diagnosis and prognosis						
Pulmonary TB	1,067 (81.0)	496 (91)	966 (79.2)	341 (73.7)	0.42 (<0.001)	
Extrapulmonary TB	400 (30.3)	97 (17.8)	354 (29.0)	153 (33.0)	2.01 (<0.001)	
Cavitation	458 (45.1)	213 (46.1)	405 (45.0)	111 (37.1)	0.96 (0.714)	
Smear-positive pulmonary TB	577 (54.2)	257 (51.9)	473 (49.2)	132 (38.7)	1.10 (0.405)	
*M. tuberculosis* culture positivity	1,102 (83.7)	486 (89.2)	979 (80.2)	402 (86.8)	0.62 (0.002)	
TB clustering	822 (81.6)	349 (77.2)	466 (51.8)	100 (27.9)	1.31 (0.050)	
Drug resistance	80 (7.3)	22 (4.5)	105 (10.7)	79 (19.7)	1.65 (0.043)	
Multidrug resistance	3 (0.3)	1 (0.2)	15 (1.5)	9 (0.9)	1.32 (0.808)	
Death¶	143 (10.8)	62 (11.4)	80 (6.6)	28 (6.0)	0.95 (0.741)	

*Stratification based on race does not include information about foreign-born blacks. Data presented as no. (%) participants except as indicated. OR, odds ratio; TB, tuberculosis; *M. tuberculosis*, *Mycobacterium tuberculosis*. †Defined according to specific zip codes. ‡Within 6 months before TB diagnosis. §Drinks alcohol daily or nearly daily. ¶Within 180 days after TB diagnosis.

The 614 HIV-infected TB patients in our study reflected a 28% rate of co-infection for US-born blacks and 20% for US-born whites (p<0.001). Rates of HIV co-infection were markedly lower for Hispanics and Asians (9.1% and 1.7%, respectively). Pulmonary disease was the predominant clinical presentation in the study population; however, extrapulmonary disease was reported more frequently for US-born blacks than for US-born whites (p<0.01).

Resistance to at least 1 anti-TB agent was recorded for 80 (7.3%) isolates from US-born blacks, compared with 22 (4.5%) isolates from US-born whites (p = 0.04). Multidrug-resistant TB represented only 1% of all cases. Mortality rate (i.e., death within 180 days after TB diagnosis) was ≈10% and was similar for US-born blacks and US-born whites.

### Clustering of Cases within Groups

Among all 4,312 cases reported during the study period, 3,578 (including enrolled and nonenrolled participants) had at least 1 positive *M. tuberculosis* culture. Isolates from 3,227 (90%) were genotyped, including 1,984 (61.5%) that matched at least 1 other isolate. A total of 242 clusters were identified, and cluster size varied from 2 to 172 patients (mean 57.2, median 27).

**Figure 2:**
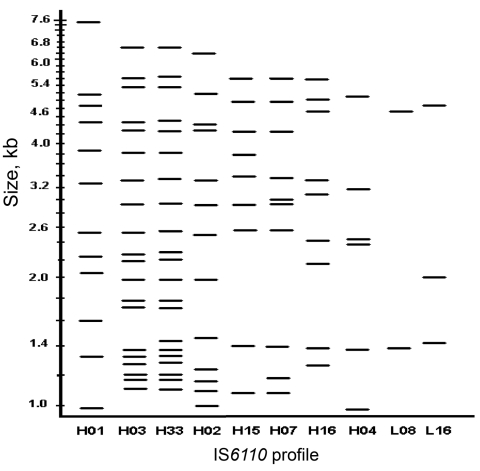
Insertion sequence (IS)*6110* profiles of the 10 largest *Mycobacterium tuberculosis* clusters, Houston Tuberculosis Initiative, Texas, 1995–2004.

Isolates of 1,765 (63%) of the 2,807 enrolled persons were clustered, including 822 (82%) of 1,007 isolates from US-born blacks and 349 (77%) of 448 isolates from US-born whites (p = 0.05). Rates of clustering were significantly lower for Hispanics and Asians (52% and 28%, respectively; p<0.01), probably underscoring a higher proportion of reactivation of latent infection in these patients. Cluster size for US-born blacks was larger (mean 69.3, median 45) than for US-born whites (mean 46.7, median 14; p<0.001). TB strains belonging to the Beijing family represented 26% and 29% of isolates from US-born blacks and US-born whites, respectively (p = 0.25). Two (H03/H33 and L16) of the 9 largest clusters containing at least 30 patients had more US-born blacks than US-born whites (Table 2, Figure 2).

**Table 2:** Characteristics of the 9 largest *Mycobacterium tuberculosis* clusters, Houston Tuberculosis Initiative, Texas, 1995–2004*

Cluster	IS*6110* copy no.	CDC spoligotype designation	Genetic group	Participant data				
				US-born black, no. (%)	US-born white, no. (%)	Hispanic, no. (%)	Asian, no. (%)	US-born black vs. white, OR (p value)
H01	12	7760 3777 7760 771	3	96 (9.5)	29 (6.4)	22 (2.44)	4 (1.11)	1.54 (0.051)
H03/H33	20/21	0000 0000 0003 771	1	109 (10.8)	31 (6.9)	8 (0.89)	2 (0.56)	1.65 (0.018)
H02	13	0000 0000 0003 771	1	77 (7.75)	28 (6.2)	20 (2.22)	1 (0.28)	1.25 (0.322)
H15	9	0000 0000 0003 771	1	25 (2.48)	7 (1.5)	6 (0.67)	0	1.62 (0.264)
H07	10	0000 0000 0003 771	1	46 (4.6)	22 (4.9)	11 (1.22)	0	0.94 (0.802)
H16	9	7777 0375 7760 771	2	28 (2.8)	8 (1.8)	5 (0.56)	0	1.59 (0.254)
H04	6	7777 7677 7760 771	2	12,(1.2)	50 (11.1)	10 (1.11)	1 (0.28)	0.10 (<0.001)
L08	2	7777 7677 7760 601	2	55 (5.5)	23 (5.1)	28 (3.11)	2 (0.56)	1.08 (0.770)
L16	3	7777 7677 7760 601	2	26 (2.6)	2 (0.4)	4 (0.44)	0 (0.00)	5.96 (0.015)

*IS, insertion sequence; OR, odds ratio.

### Multivariate Logistic Regression Analysis

In our final model (Table 3), factors independently associated with black race among TB patients included younger age, fewer years of education, inner city residence, use of public transportation, prison history, renal disease, and HIV seropositivity. US-born blacks were significantly less likely than U.S.-born whites to report current homelessness, smoking, alcohol abuse, injection drug use, and same-sex behavior. Although male sex, not being married, *M. tuberculosis* culture positivity, employment, and extrapulmonary disease were significant in the univariate analysis, they were no longer significant in the full model and therefore were dropped from the final parsimonious model. Because including certain covariates significantly decreased the sample size (and the representativeness of the dataset), we developed a second model (data not shown) by adding drug resistance and clustering to the final model. In this model, drug resistance remained significantly higher for US-born blacks than for US-born whites (odds ratio [OR] 1.76; 95% confidence interval [CI] 1.02–3.04, p = 0.04); however, clustering did not remain significant (OR 1.13, 95% CI 0.82–1.54, p = 0.46).

**Table 3:** Factors associated with black race in multivariate analysis in US-born TB patients, Houston Tuberculosis Initiative, Texas, 1995–2004*

Risk factor	OR	95% CI	p value
Age	0.98†	0.97–0.99	<0.001
Years of education	0.93†	0.89–0.96	<0.001
Inner city residence‡	1.44	1.13–1.85	0.003
Use of public transportation	2.44	1.91–3.11	<0.001
Current homelessness	0.48	0.33–0.68	<0.001
Current smoking	0.54	0.42–0.70	<0.001
Alcohol abuse§	0.63	0.50–0.80	0.001
Injection drug use¶	0.30	0.16–0.57	<0.001
Same-sex sexual behavior	0.28	0.19–0.41	<0.001
Prison history	1.42	1.06–1.91	0.020
Renal disease	2.61	1.38–4.92	0.003
HIV seropositivity	1.89	1.37–2.61	<0.001

*TB, tuberculosis; OR, odds ratio; CI, confidence interval. †ORs per additional year at risk. ‡Defined according to specific zip codes. §Drinks alcohol daily or nearly daily. ¶Within 6 months before TB diagnosis.

## Discussion

Although the incidence rate of TB in the United States during 2007 was the lowest recorded since national reporting began in 1953, the decline has slowed from an average of 7.1% per year (1993–2000) to an average of 3.8% per year (2001–2007) (*1*). Our population-based study of TB in a large US metropolitan city emphasized the epidemiologic, molecular, and clinical characteristics of US-born blacks. During the 9-year study period, the overall incidence of TB in Harris County, Texas, decreased dramatically; however, the incidence among blacks remained fairly stable (22.6–25.2 cases per 100,000) over the past 5 years of the study. This incidence is 4–5× the US national TB incidence rate (*1*). Numerous factors have been postulated to explain the disproportionately high TB rate for blacks, including socioeconomic characteristics, biasing presence of comorbidities, and genetics (*15*,*16*).

Our evaluation of socioeconomic characteristics demonstrated that younger age, fewer years of education, use of public transportation, and inner city residence were independently associated with black race among TB patients. These associations support the concept that TB remains predominantly a disease of disadvantaged and marginalized persons (*17*,*18*). Contrary to findings recently reported (*18*), our population-based study showed that US-born whites with TB were more likely than US-born blacks with TB to be homeless. In addition, certain clinical conditions such as renal disease and HIV seropositivity were more likely in US-born blacks with TB.

*M. tuberculosis* isolates from US-born blacks were more likely to be resistant than were those from US-born whites, which was in accordance with previously reported findings (*19*). However, foreign-born persons, particularly Hispanics and Asians, still have the most drug-resistant TB in Houston (*20*). Among our study population, death from any cause within 6 months after TB diagnosis reported was ≈10%. We found no difference in TB-associated mortality rates between US-born blacks and whites.

TB strains clustered in a high proportion of both US-born blacks and US-born whites (81.6% and 77.2%, respectively; p = 0.46). We previously demonstrated that ethnicity was not a significant covariate for strain clustering after adjustments for factors related to socioeconomic status (*21*). Isolates belonging to the Beijing genotype family clustered in 26% and 29% of US-born blacks and US-born whites, respectively (p = 0.25). This genetically highly conserved family, reported worldwide but particularly prevalent in Asia and the territories of the former Soviet Union (*22*,*23*), is frequently associated with large TB outbreaks, increased virulence, and multidrug resistance (*24*,*25*).

One of the most striking findings of our study is the markedly large size of clusters. Previous US-based studies had reported cluster sizes not exceeding 30–78 patients each (*6*,*8*,*9*). We reported 9 clusters with >30 patients each and 3 clusters with >100 patients each, including 1 with a predominance of blacks. This may be due to the longer surveillance period used here, as well as to endemic spread of highly transmissible strains, including several of the Beijing family. We estimated that at least 54% (1,984 – 242 [of 3,227]) of TB cases in Houston resulted from recent infection that had progressed to active disease during the 9-year study period (*6*). We believe this estimate is conservative because a strict definition of identical IS*6110* RFLP bands was used for clustering, and several RFLP patterns in our study were similar but with ±1 band (Figure 2).

Several TB genotypes remain endemic to Houston, particularly in minorities such as US-born blacks. A high proportion of endemic strains of *M. tuberculosis* remain actively transmitted within this population. Further research on the dynamics of the disease, including possible delays in care-seeking behavior or diagnosis in this racial group, is of paramount importance to achieve the national goal for TB elimination.
